# De Winter presentations and considerations: a case series

**DOI:** 10.1186/s13256-022-03604-3

**Published:** 2022-10-11

**Authors:** Bahram Shahri, Mohammad Vojdanparast, Faeze Keihanian, Ali Eshraghi

**Affiliations:** 1 Interventional Cardiologist, Faculty of Medicine, Ghaem Hospital, Mashhad University of Medical Sciences, Mashhad, Iran; 2 Interventional Cardiologist, Faculty of Medicine, Imam Reza Hospital, Mashhad University of Medical Sciences, Mashhad, Iran; 3 Echocardiologist, Faculty of Medicine, Imam Reza and Ghaem Hospital, Mashhad University of Medical Sciences, Mashhad, Iran; 4 Pharmaceutical Research Center, Mashhad University of Medical Sciences, Mashhad, Iran

**Keywords:** De Winter, Electrocardiogram, ST-elevation myocardial infarction

## Abstract

**Background:**

The electrocardiogram has a critical role in the diagnosis and risk assessment of patients presenting with chest pain in the emergency ward.

**Case presentation:**

We present 11 Iranian patients with diagnosis of de Winter referred to our center. Right coronary artery involvement was seen in four cases, left circumflex artery in three cases, proximal left anterior descending artery in two cases, and middle left anterior descending artery in seven cases. We present the case of a 52-year old Iranian male patient in detail.

**Conclusion:**

Recognizing the electrocardiogram of de Winter as an ST-elevation myocardial infarction equivalent in cases with suspected acute infarction is very important.

## Introduction

Various presentations of chest pain to the emergency ward is always challenging in clinical practice. They can range from being harmless to cardiogenic shock and arrest [1], and can result from benign conditions such as noncardiogenic causes. The electrocardiogram (ECG) has a critical role in the diagnosis and risk assessment of patients. Some ECG findings are higher risk and are correlated with dangerous outcomes that need urgent management [1]. In the lack of ST elevation in ECG, there may be some patterns that require emergent angiography [2]. Understanding de Winter ECG patterns as an ST-elevation myocardial infarction (STEMI) equivalent in cases with suspected acute myocardial infarction is very important, despite its rare incidence, as it indicates an immediate need for emergent revascularization [3].

We herein report a case series of patients with a diagnosis of de Winter syndrome, their presentations and ECG changes, and our therapeutic modalities.

## Case presentation

In this study, we present 11 Iranian patients with a diagnosis of de Winter referred to our center in Imam Reza Hospital during 2017–2018. The mean age of the patients was 51.91 ± 16.35 (30–82) years. Five of the patients were female and six were male. Table1 presents the patients’ characteristics.

**Table 1:** Characteristics of patients with de Winter syndrome

Patient no.	Age (years)	Gender	Diabetes mellitus	Hypertension	Smoker
1	63	Female	+	−	−
2	34	Male	−	−	+
3	52	Male	−	−	+
4	62	Male	−	−	−
5	46	Male	−	−	−
6	30	Female	+	−	−
7	69	Female	+	+	−
8	82	Female	+	+	−
9	56	Male	+	−	−
10	34	Female	−	−	−
11	43	Male	−	−	−

Right coronary artery involvement was seen in four cases (4/11), left circumflex artery in three cases (3/11), proximal left anterior descending artery in three cases (3/11), and middle left anterior descending artery in seven cases (7/11). Table2 presents the angiographic findings of patients.

**Table 2:** Angiographic findings of patients with de Winter syndrome

Patient no.	Angiographic involvement						
	Significant proximal LAD	Significant mid part LAD	Significant ostial LCX	Significant proximal LCX	Significant proximal RCA	Significant mid part RCA	Significant distal RCA
1	−	+	+	−	+	−	−
2	+	−	−	+	−	−	−
3	−	+	−	−	−	−	+
4	−	+	−	−	−	+	−
5	+	−	−	−	−	−	−
6	−	+	−	−	−	−	−
7	−	+	−	−	−	−	−
8	−	+	−	−	+	−	−
9	−	+	−	−	−	−	−
10	+	−	−	−	−	−	−
11	−	−	+	−	−	−	−

*LCX* left circumflex artery, *LAD* left anterior descending artery, *RCA* right coronary artery

The variation in age in our case series was high. We report a 30-year-old diabetic female with proximal LAD involvement without previous positive familial history, as well as two 34-year-old patients, one of whom had no cardiovascular risk factor. In our hospital, all patients were undergoing emergent percutaneous coronary intervention (PCI) after transfer to the catheterization laboratory. After catheterization, pharmacological treatment was prescribed according to guidelines.

Here we introduce one of the patients, a 52-year-old male was referred to the emergency department of Imam Reza Hospital, Mashhad University of Medical Sciences, due to chest pain for 2 hours before admission. He had no documented comorbidity.

**Fig. 1:**
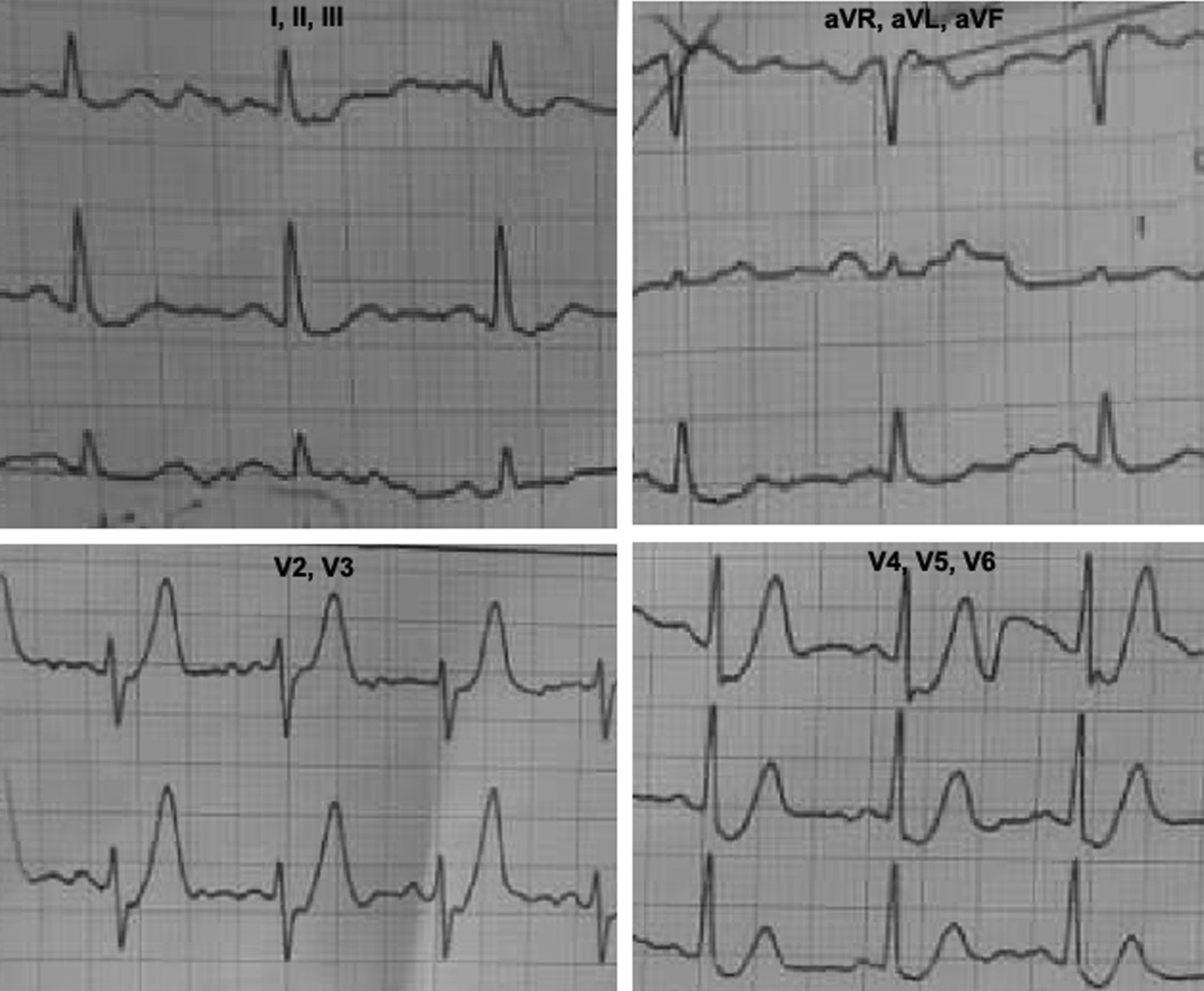
Electrocardiography of patient at time of admission

**Fig. 2:**
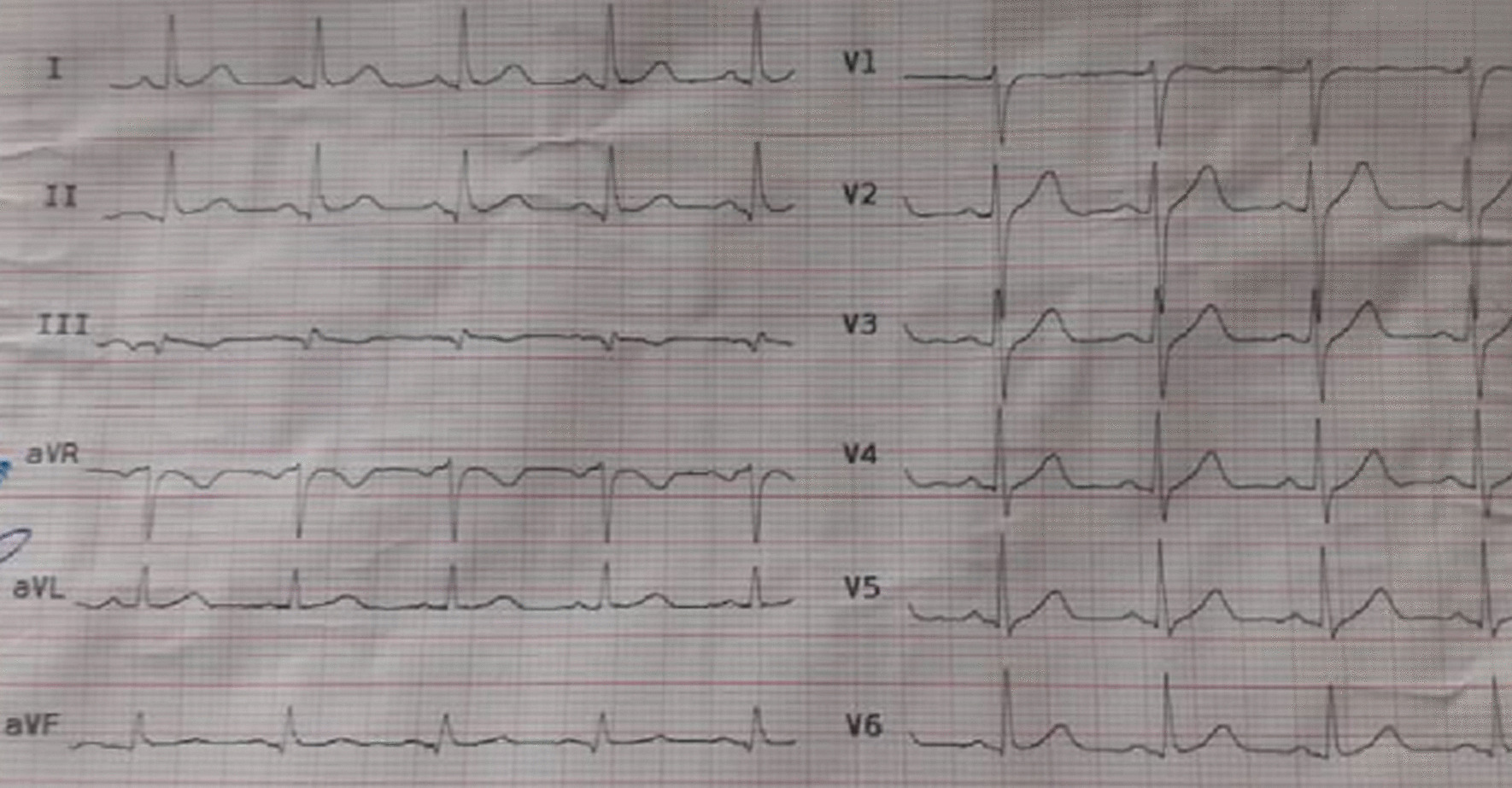
Post-percutaneous coronary intervention electrocardiography

He was a current smoker with a 25-year-history of smoking 20 cigarettes per day. He was fully conscious and had a regular heart rhythm (100 beats/minute) with normal heart sounds. No cardiac murmurs were heard on auscultation. The results of the lung and abdomen examinations were unremarkable. Five minutes after his admission, an emergency ECG (Fig. 1) showed sinus rhythm and up-sloping ST-segment depression in leads V2–V6. Laboratory data showed elevated creatinine kinase (CK)-MB and troponin I. Treatment with Plavix  (600 mg) and chewable aspirin (300 mg) was performed immediately. Before transferring the patient to the catheterization laboratory, he had two episodes of ventricular fibrillation terminated by direct-current (DC) shock. He underwent emergent catheterization within 15 minutes of admission. Angiographic data revealed significant (99%) mid-part stenosis in the left anterior descending artery (LAD), significant (95%) ostial stenosis of the second diagonal branch (bifurcation lesion), and significant distal stenosis in the right coronary artery (RCA). Primary PCI on bifurcation stenosis was performed (Fig. 2). He was discharged in good condition and recommended for close medical follow-up.

## Discussion and conclusions

Presenting with chest pain and no ST segment elevation, such as refractory angina, non-ST-elevation MI (NSTEMI) with unstable hemodynamic, and so on, needs prompt invasive intervention. There are also conditions equivalent to ST segment elevation, such as newly developed left bundle branch block (LBBB), Wellens syndrome, and de Winter T waves [4, 5]. In our series, we present a young patient with de Winter, which has not been reported in the literature before. We also report cases with different presentations and different territories of vessel involvement.

De Winter syndrome is an ECG pattern related to acute occlusion of the left anterior descending artery (LAD), which was first described by de Winter* et al*. in 2008. The incidence rate of de Winter syndrome is approximately 2% of all patients with acute anterior myocardial infarction, which is relatively rare, but it still requires attention from clinicians [6, 7]. De Winter syndrome is an acute coronary syndrome in which the left anterior descending artery is the most involved vessel. However, it is also related to the occlusion of other arteries [8].

The specific ECG patterns of de Winter syndrome are as follows [6]: (a) 1–3 mm up-sloping ST-segment depression at the J point in leads V1–V6 that continue into tall, positive symmetrical T waves; (b) QRS complex is usually narrow or only slightly widened; (c) in some patients, there is an abnormal precordial R-wave progression; (d) 1–2 mm ST-segment elevation in aVR lead in most cases. T-wave spikes may also be indicative of acute coronary syndromes because this finding may be caused by the deterioration in early blood flow [9, 10]. A de Winter ECG also shows long and distinct T waves, but unlike hyper-acute T waves, T spikes in these patients are fixed and the lesion continues until revascularization is accomplished [11]. ST segment depression is uncertain, and a possible hypothesis is that retrograde filling of the LAD with collateral blood vessels and prolonged repolarization of the endocardium causes an increased repolarization vector in the same direction [12].

Ideally, the presence of a de Winter T wave ECG should be treated as urgent as STEMI, with catheter laboratory activation for coronary angiography and possible stenting [4]. Thrombolysis was initially avoided because a de Winter T wave ECG is currently not an indication for fibrinolysis even in the latest guidelines, and there was no clear-cut evidence of acute coronary occlusion [13]. Regardless of the debate, the most important issue is to recognize this ECG pattern and prevent a delay in management, as this increases the total ischemic time, which is related to higher mortality in STEMI. However, whether the same applies to de Winter T wave ECGs is unclear [14].

Our findings showed that coronary artery occlusion was seen in more than 40% of cases in vessels other than LAD and can be varied. Another important finding of our case series was the different presentations of this pattern and the younger age of patients. Considering ECG as a simple and available tool for the diagnosis of de Winter and its specific pattern is very important to correctly diagnose such cases.

## Data Availability

Data sharing is not applicable to this article, as no datasets were generated or analyzed during the current study.
